# Prevalence of Acute Alcohol Use in Traumatic Brain Injury Patients During the COVID-19 Pandemic: A Retrospective Analysis From Queens, New York

**DOI:** 10.7759/cureus.58928

**Published:** 2024-04-24

**Authors:** Pemla Jagtiani, Tirone Young, Wasil Ahmed, Alex Devarajan, Zachary L Hickman, Salazar Jones

**Affiliations:** 1 School of Medicine, State University of New York Downstate Health Sciences University, Queens, USA; 2 Medical School, Icahn School of Medicine at Mount Sinai, New York, USA; 3 Neurosurgery, Icahn School of Medicine at Mount Sinai, New York, USA; 4 Neurological Surgery, Mount Sinai Hospital, New York, USA

**Keywords:** epidemiology, tbi, covid-19, alcohol, trauma

## Abstract

Background

This study investigates the impact of New York’s relaxed alcohol consumption policies during the coronavirus disease (COVID-19) pandemic on alcohol-related traumatic brain injuries (TBIs) among patients admitted to a Level 1 trauma center in Queens. Given the limited research available, this study critically explores the link between public health policies and trauma care. It aims to address a significant gap in the literature and highlight the implications of alcohol regulations during global health emergencies.

Methodology

A retrospective analysis was conducted among trauma patients from 2019 to 2021. The study period was divided into the following three periods: pre-lockdown (March 7, 2019, to July 31, 2019), lockdown (March 7, 2020, to July 31, 2020), and post-lockdown (March 7, 2021, to July 31, 2021). Data on demographics, injury severity, comorbidities, and outcomes were collected. The study focused on assessing the correlation between New York’s alcohol policies and alcohol-related TBI admissions during these periods.

Results

A total of 1,074 admissions were analyzed. The study found no significant changes in alcohol-positive patients over the full calendar years of 2019, 2020, and 2021 (42.65%, 38.91%, and 31.16% respectively; p = 0.08711). Specifically, during the lockdown period, rates of alcohol-positive TBI patients remained unchanged, despite the relaxed alcohol policies. There was a decrease in alcohol-related TBI admissions in 2021 compared to 2020 during the lockdown period.

Conclusions

Our study concludes that New York’s specific alcohol policies during the COVID-19 pandemic were not correlated with an increase in alcohol-related TBI admissions. Despite the relaxation of alcohol consumption laws, there was no increase in alcohol positivity among TBI patients. The findings suggest a complex relationship between public policies, alcohol use, and trauma during pandemic conditions, indicating that factors other than policy relaxation might influence alcohol-related trauma incidences.

## Introduction

The first U.S. COVID-19 case was confirmed on January 30, 2020, prompting the White House to declare a national emergency and enforce social distancing guidelines [1]. This led states to implement their own measures, such as mandatory mask-wearing and restrictions on gatherings. As the pandemic progressed, these diverse local, federal, and international responses had profound socioeconomic and geopolitical effects. The necessity to balance individual freedoms with public health regulations triggered widespread economic disruptions [2,3]. Global markets faced severe downturns, businesses cut back or halted operations, and job losses escalated across crucial sectors, including manufacturing, agriculture, and education [4,5]. Additionally, the pandemic exerted a heavy toll on mental health worldwide, with long-lasting repercussions on public well-being and economic stability [6].

Amid the changes in public health policy, there were varied governmental responses concerning the sale and consumption of alcohol [7-9]. Several studies have investigated the effect of public policy in reducing alcohol-related trauma [10,11]. Lange et al. suggested that relaxed alcohol control policies would lead to increases in heavy alcohol consumption, which, in turn, can lead to a greater number of unintentional injuries. Lange et al. suggested precautionary measures by way of public policy ought to be taken to reduce easily preventable alcohol-related intentional injuries [10]. Stockwell et al. similarly argued for more restrictive alcohol policies and increased taxation to reduce the trauma burden within healthcare services [11].

While there are studies examining a rise in alcohol-related traumas during the COVID-19 pandemic, there is minimal data examining alcohol use in patients with traumatic brain injuries (TBIs) and how this might be related to alcohol-specific policies put in place during the pandemic. Related studies primarily concentrated on analyzing alcohol’s impact on mood [12,13] during this period were based internationally [14,15], conducted systematic reviews of existing literature [16], or were exclusively qualitative [17]. This is the first original article focusing on the specific experience of a single center. Considering the global burden of TBI, with over 69,000,000 individuals affected annually, and its position as a leading traumatic contributor to death and disability worldwide, the significance of studying this intersection is clear [18]. Data from the World Health Organization estimates that TBI is the main cause of as much as half of all trauma-related deaths globally [19]. Given the well-established link between alcohol use and TBI, the implications of COVID-19-era alcohol policies on this crucial health issue merit close examination [20,21].

There are several unique variables related to the New York City (NYC) experience. NYC was hardest hit by the first wave of COVID-19 infections. NYC lost its daily influx of commuters and tourists due to the lockdown which affected the total population susceptible to trauma. Among NYC residents, there was also a documented outmigration of 5% of residents between March 1, 2020, and May 1, 2020 [22]. NYC mechanisms of trauma do not typically involve high-speed motor accidents to which alcohol is strongly related as most NYC individuals likely walk or use public transportation. While other local governments were restricting or banning alcohol sales, NYC relaxed control policies by still permitting alcohol to be sold to-go. We chose to examine the impact, if any, of the COVID-19 lockdown and subsequent relaxed alcohol policies on alcohol-related TBIs presenting to a busy Level 1 trauma hospital.

## Materials and methods

This study, involving a retrospective review of de-identified patient records, adhered to ethical standards for research with human subjects. All data were anonymized to ensure confidentiality and privacy. The research received approval from the BRANY Institutional Review Board, confirming compliance with ethical guidelines.

This retrospective analysis was conducted among patients who presented to a Level I trauma center in Queens, New York (Elmhurst Hospital Center). This study included patients with TBI presenting to the emergency department (ED) aged ≥18 years admitted from January 1, 2019, to December 31, 2021. Only patients with positive alcohol test results at the time of admission were included in the study. Alcohol use in patients was compared across the full calendar year of 2019 to 2021 to account for seasonal variation in trauma admissions. Three more focused periods were used to compare rates of alcohol use before, during, and after the lockdown period: pre-lockdown (March 7, 2019, to July 31, 2019), lockdown (March 7, 2020, to July 31, 2020), post-lockdown (March 7, 2021, to July 31, 2021). These focused time periods were used because March 16, 2020, marks the date of the executive order to close nonessential businesses and start to-go alcohol sales for restaurants, and July 17, 2020, marks the date the sale of alcohol was restricted in restaurants to people who did not also buy food. The same dates were used across the three years to account for seasonal changes in alcohol use and traumatic injury.

Demographic data was collected to examine potential changes in the profile of TBI patients or hospital course. The following demographic data were collected: sex, race, age, mechanism of injury (accident, assault, bicycle, fall, gunshot wound, motorcycle crash, motor vehicle crash, pedestrian, stab wound, suicide), injury severity score (ISS, 1-9, 10-15, ≥16), presence of comorbidities (mental illness, alcoholism, substance abuse, diabetes mellitus, hypertension, chronic renal failure, anticoagulant therapy, current smoker, disseminated cancer, bleeding disorder, cirrhosis, advanced directive limiting care, functionally dependent health status), hospital length of stay (LOS), intensive care unit (ICU) stay, ventilator use, days on the ventilator, in-hospital mortality, and different types of procedures (intracranial pressure (ICP) monitor, craniotomy/craniectomy, tracheostomy/ percutaneous endoscopic gastrostomy (PEG), other).

Statistical analyses

The number of patients screened and those positive for alcohol presenting with TBI were examined as percent by COVID-19 time period (pre, during, or post).

The analysis of the demographic, clinical, and procedural variables across the three time periods was conducted through univariate statistical tests. Categorical variables were assessed using Pearson chi-square tests, with continuity corrections applied when necessary. If a particular categorical variable had at least 25% of expected outcomes with n ≤ 5, Fisher’s exact tests were employed instead. For continuous variables, two-tailed paired t-tests were employed if the data followed a normal distribution. However, when the assumption of normality was not met, the Wilcoxon signed-rank test, a non-parametric test, was utilized to assess differences. A significance level of α = 0.05 was used to conduct all statistical analyses.

## Results

A total of 1,074 patients were admitted across the three-year study period with the following breakdown: 32.3% in 2019, 30.6% in 2020, and 37.1% in 2021. While there was an absolute decrease in TBI admissions during the COVID-19 pandemic (p = 0.0279), there were no significant changes in alcohol-positive patients between these periods (42.7% vs. 38.9% vs 31.2%; p = 0.08711) (Table 1).

**Table 1:** Overall rates of alcohol-positive TBI patients by year (N = 1,074). N = total number of TBI patients. * = Year-by-year analysis revealed a statistically significant difference only between 2020 and 2021 in terms of number of TBI patients. TBI = traumatic brain injury

	2019	2020	2021	P-value
# TBI patients	347	329	398	0.0279*
+ Alcohol Screening	148 (42.7%)	128 (38.9%)	124 (31.2%)	0.08711

**Figure 1:**
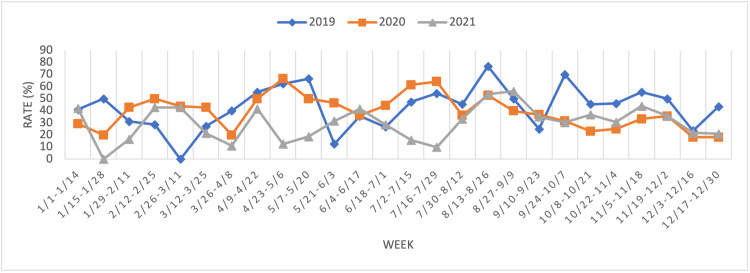
Alcohol-positive rates by week.

Figure 1 shows the rates of admissions in which patients tested positive on alcohol screening, every two weeks and for each period year. Following the national stay-at-home guidelines of March 16, 2020, there was an immediate decrease in rates for a couple of weeks (March 12, 2020, to April 8, 2020), followed by an increase for the next four weeks (March 26, 2020, to May 6, 2020). The first phase of re-opening, set on April 27, 2020, coincides with a drop in rates (April 23, 2020, to May 6, 2020) that was sustained for a couple of months (April 23, 2020, to June 17, 2020).

A total of 373 patients were admitted with the following breakdown: 37.0% in pre-lockdown (March 7, 2019, to July 31, 2019), 25.7% in lockdown (March 7, 2020, to July 31, 2020), 37.3% in post-lockdown (March 7, 2021, to July 31, 2021). There was a statistically significant decrease in alcohol-related TBI admissions during the lockdown (p = 0.0079) in line with other major trauma centers. Between these periods, there were no significant differences in the number of patients screened for alcohol (77.54% vs. 90.63% vs. 88.49%; p = 0.13640). Patients who tested positive on alcohol screening showed a notable change across the same periods (39.9% vs. 49% vs. 28.4%; p = 0.02241). However, a year-by-year analysis revealed that a statistically significant difference in alcohol screening results was only observed between 2020 and 2021 (Table 2).

**Table 2:** Overall rates of alcohol-positive TBI patients before, during, and after COVID-19 lockdown (N = 373). Pre-lockdown: March 7, 2019, to July 31, 2019; lockdown: March 7, 2020, to July 31, 2020; post-lockdown: March 7, 2021, to July 31, 2021. * = Year-by-year analysis revealed a statistically significant difference only between pre-lockdown versus lockdown, and lockdown versus post-lockdown in terms of the number of TBI patients. ** = Year-by-year analysis revealed a statistically significant difference only between lockdown and post-lockdown in terms of alcohol positivity. COVID-19 = coronavirus disease 2019; TBI = traumatic brain injury

	Pre-lockdown	Lockdown	Post-lockdown	P-value
# TBI patients	138	96	139	*0.0079
Total screened	107	87	123	0.13640
+ Alcohol	55 (39.9%)	47 (49.0%)	35 (28.4%)	**0.02241

In evaluating the characteristics and outcomes of patients with positive alcohol screening, a total of 142 patients were identified within the more focused periods. The gender distribution within this cohort was predominantly male across the pre-lockdown (43, 78.2%), lockdown (46, 97.87%), and post-lockdown periods (30, 85.7%, p = 0.013). The “Other” racial category constituted the largest portion of patients who disclosed their racial identities, making up 60% in the pre-lockdown, 76.6% during lockdown, and 68.6% post-lockdown. Across all three periods, approximately half of the patients were at least 30 years old, with almost a third falling into the young adult category (29 years of age or under). The predominant mechanism of injury before, during, and after the pandemic was falls, although assaults stood out as significant precursors to TBI. When categorized based on our predefined ranges (1-9, 10-15, >16), the injury severity scores were evenly distributed in 2019. However, in 2020 and 2021, there was an increase in traumatic scores, although they did not show statistically significant differences (p = 0.668). Alcoholism, hypertension, and diabetes mellitus were the most common comorbidities. The median length of stay remained shorter than five days in all three periods, with about two-thirds of patients in each cohort requiring ICU stays. Ventilator use varied across study periods, with patients in 2020 requiring sustained ventilation. Regarding medical interventions, less than 5% of the total patient population required intracranial monitoring procedures. In contrast, a substantial portion (38.7%) underwent one or more surgical procedures of varying types (Table 3). Tracheostomy and percutaneous endoscopic gastrostomy (PEG) procedures increased through 2020, subsequently declining to 14.29% in 2021. Surgical interventions, including ICP monitor placement and craniotomy, exhibited fluctuations throughout the study periods.

**Table 3:** Characteristics and outcomes of patients with positive alcohol screening results (N = 142). * = Pre-lockdown: March 7, 2019, to July 31, 2019; † = lockdown: March 7, 2020, to July 31, 2020; ‡ = post-lockdown: March 7, 2021, to July 31, 2021. Bolded p-value indicates statistical significance. GSW = gunshot wound; MCC = motorcycle crash; MVC = motor vehicle crash; LOS = length of stay; ICU = intensive care unit; ICP = intracranial pressure; PEG = percutaneous endoscopic gastrostomy

Characteristics, n (%)		Pre-lockdown, N = 55 (2019)*		Lockdown, N = 47 (2020)†		Post-lockdown, N = 35 (2021)‡		P-value
		n	%	n	%	n	%	
Sex								0.013
	Male	43	78.18	46	97.87	30	85.71	
	Female	12	21.82	1	2.13	5	14.29	
Race								0.052
	Black	2	3.64	1	2.13	4	11.43	
	Asian	5	9.09	1	2.13	0	0.00	
	White	10	18.18	4	8.51	7	20.00	
	Other	33	60.00	36	76.60	24	68.57	
	Unknown	5	9.09	5	10.64	0	0.00	
Age range, years								0.427
	18–20	1	1.82	1	2.13	2	5.71	
	21–34	16	29.09	13	27.66	14	40.00	
	35–64	30	54.55	29	61.70	18	51.43	
	>65	8	14.55	4	8.51	1	2.86	
Mechanism of injury								0.413
	Accident	0	0.00	0	0.00	0	0.00	
	Assault	9	16.36	11	23.40	12	34.29	
	Bicycle	5	9.09	7	14.89	2	5.71	
	Fall	30	54.55	22	46.81	11	31.43	
	GSW	1	1.82	0	0.00	0	0.00	
	MCC	1	1.82	4	8.51	4	11.43	
	MVC	2	3.64	1	2.13	2	5.71	
	Pedestrian	5	9.09	1	2.13	3	8.57	
	Stab Wound	1	1.82	1	2.13	1	2.86	
	Suicide	1	1.82	0	0.00	0	0.00	
Injury Severity Score								0.668
	1–9	18	32.73	12	25.53	9	25.71	
	10–15	18	32.73	17	36.17	9	25.71	
	>16	19	34.55	18	38.30	17	48.57	
Comorbidities								
	Mental illness	2	3.64	3	6.38	1	2.86	0.699
	Alcoholism	17	30.91	17	36.17	15	42.86	0.513
	Substance abuse	1	1.82	6	12.77	4	11.43	0.089
	Diabetes mellitus	7	12.73	6	12.77	2	5.71	0.517
	Hypertension	10	18.18	8	17.02	3	8.57	0.432
	Chronic renal failure	0	0.00	2	4.26	0	0.00	0.143
	Anticoagulant therapy	1	1.82	0	0.00	0	0.00	0.472
	Current smoker	3	5.45	5	10.64	8	22.86	0.042
	Disseminated cancer	0	0.00	1	2.13	0	0.00	0.381
	Bleeding disorder	0	0.00	0	0.00	1	2.86	0.230
	Cirrhosis	1	1.82	2	4.26	1	2.86	0.767
	Advanced directive limiting care	3	5.45	0	0.00	0	0.00	0.102
	Functionally dependent health status	0	0.00	2	4.26	0	0.00	0.143
LOS, median (IQR), days		3.43	(0-30.71)	2.4	(0-45.3)	2.89	(0-20.48)	0.731
ICU stay		35	63.64	32	68.09	25	71.43	0.735
Ventilator use		17	30.91	17	36.17	6	17.14	0.162
Days on ventilator								0.434
	1–9	11	20.00	10	21.28	4	11.43	
	10–15	5	9.09	4	8.51	1	2.86	
	>16	1	1.82	3	6.38	1	2.86	
In-hospital mortality		6	10.91	4	8.51	2	5.71	0.695
Procedures								
	ICP monitor	5	9.09	2	4.26	1	2.86	0.399
	Craniotomy/Craniectomy	4	7.27	6	12.77	1	2.86	0.268
	Tracheostomy/PEG	12	21.82	15	31.91	5	14.29	
	Other surgical procedures	18	32.73	21	44.68	10	28.57	

## Discussion

This retrospective cohort examined the differences in alcohol use among TBI patients admitted during the COVID-19 pandemic in 2020 compared to the previous and subsequent years. We noted an absolute decrease in TBI admissions during the COVID-19 pandemic with no significant difference in the proportion of alcohol positivity. Several other reports have examined the relationship between alcohol and TBI admissions during the COVID-19 pandemic with conflicting results. Thomas et al. studied the volume of trauma center admissions after a stay-at-home order, namely, patients who presented with a pre-existing history of alcohol abuse. While they noted an increase in the proportion of trauma patients with chronic alcohol use and head injury, they did not explicitly examine acute alcohol intoxication and its relationship to TBI [23]. Rault et al. noted an overall decrease in TBI admissions during the COVID-19 pandemic, but a relative increase in alcohol-related TBI. Their study, however, was severely limited by a small sample size, as their study examined patients presenting to the ICU at only two trauma centers in Normandy, France [15]. Laufer et al. reported a decrease in alcohol blood levels in TBI patients during the pandemic, attributed to decreased mobility and fewer social events. They also report a decrease in the number of patients presenting with mild TBI during the pandemic but do not comment on the proportion of TBI patients with acute intoxication [24].

Although limited in the literature, the varied experiences seen in alcohol-related TBI admissions are paralleled when examining the wider area of trauma admissions. Several studies have reported an increase in alcohol positivity among trauma patients [1,25-28]. One such study by Brown et al. emphasized the impact of the lockdown in causing mental and financial distress that can elucidate binge drinking [26]. This study was conducted at a major trauma center in London and revealed a significantly higher proportion of alcohol-related trauma during the lockdown (31.4%) versus before the lockdown (19.6%). Similarly, a study conducted at a Level 1 rural trauma center in West Virginia showed 15.5% of injured drivers being alcohol-positive pre-COVID-19 and 17.4% being alcohol-positive during COVID-19 [29]. While there were across-the-board increases in alcohol-related trauma, the total number of trauma admissions during the COVID-19 pandemic decreased. Therefore, the reported increases in alcohol-related traumas were increases in the relative proportion of all traumas. As such, the data could also be explained by the COVID-19 pandemic more greatly suppressing non-alcohol-related traumas than alcohol-related trauma.

By contrast, several studies found no significant difference in alcohol-related traumas during the COVID-19 pandemic. In New Jersey, Zhai et al. noted a rise in the prevalence of diagnosed alcohol use in trauma patients, but the rates of acute alcohol positivity remained the same. Overall, 34% of trauma patients were alcohol-positive the year before COVID-19 versus 33% during COVID-19, and this difference did not reach statistical significance [30]. Young et al. conducted a retrospective analysis of 11 trauma centers in Southern California and found no difference in alcohol positivity or blood concentration after stay-at-home orders were issued. The post-stay-at-home order group had an alcohol-positive rate of 29.7%, similar to the pre-stay-at-home order group and control group pre-COVID-19 [31]. A similar conclusion was found by Sun et al. in their retrospective review of patients from the TriNetX database. After stay-at-home orders were placed, they observed a 0.76% increase in alcohol-related presentations to the ED, but this did not reach statistical significance [32].

Our study noted a higher incidence of craniotomies, tracheostomies, and PEGs during the 2020 period. Despite comparable mechanisms of injury and injury severity scores across the three years, this trend raises questions about potential mediating factors. One clear external factor during 2020, the COVID-19 pandemic, could have played a role in influencing patient outcomes and procedural decisions. Although our data lacks explicit records of patients’ COVID-19 status, it is plausible that the concurrent presence of the virus may have contributed to the higher percentage of patients requiring tracheostomies, PEGs, and even craniotomies. Patients with COVID-19, especially those with severe manifestations, often experience respiratory distress and may necessitate prolonged intubation [33]. This, in turn, could increase the likelihood of tracheostomies and potentially contribute to the need for craniotomies in cases where the Glasgow Coma Scale is compromised due to factors such as sepsis or extended intubation [34-38]. The absence of explicit COVID-19 status records limits the depth of our conclusions. However, acknowledging the potential influence of the pandemic on procedural decisions is essential for a comprehensive understanding of the observed trends.

We also examined whether the relaxation of alcohol consumption laws impacted the rate of alcohol-related TBI admissions. The ongoing reduction in TBI alcohol positivity is evident through a decrease in the absolute number of alcohol-related TBI traumas, along with an increase in non-alcohol-related TBIs in 2021. The exact mechanism is not clear and cannot be determined by changes in the mechanism or demographics of patients. Therefore, further research is needed. While there was a rise in alcohol positivity right after March 2020, it does not appear related to the relaxing of laws around alcohol consumption. There are several potential explanations for why we did not see any significant differences in alcohol-related TBI admissions during the lockdown period. By a March 16, 2020, New York executive order, nonessential businesses were closed but permitted “to-go” alcohol sales from bars and restaurants as “essential” services along with public transportation [1]. With alcohol still being sold to-go, it is possible alcohol consumption and TBI-related activities were less affected. Another factor could be the easily accessible public transportation. Individuals who were drinking at home or grabbing alcohol to-go during the pandemic were likely walking or using public transportation, rather than their own vehicles. Throughout the 2019-2021 period, motor vehicle collisions remained a very uncommon mechanism of injury as would be seen in a more rural setting.

Yet, public health policy can reduce alcohol-related trauma admissions. The following studies found stricter laws during the pandemic to be beneficial in reducing the number of trauma victims. In a large rural hospital in South Africa, the government enforced strict bans on the sale and distribution of alcohol, completely banning alcohol. This study found a 59-69% decrease in trauma volume when the first complete ban was initiated. Moreover, when partial bans were reinstated, there was an 83-90% increase in trauma volume, which then dropped again by 39-46% with the reinstatement of the second complete ban. The establishment of strict policies was likely beneficial in South Africa because South Africa reports a generally larger percentage of alcohol-related injuries presenting to ED, 36-79%, compared to an international percentage of 15% [39]. Another study from South Africa reported similar results with the prohibition of alcohol sales during the lockdown. As a result of these policies, there was a sharp decline in unnatural deaths from 800-1,000/week to 400/week during the lockdown [40]. Their study is unable to separate alcohol ban from other confounders, though the authors do suggest a new public health policy in which alcohol is restricted and encourage this implementation in other African states as well. In Tamil Nadu, South India, a similar trend was noticed with the closure of liquor shops during the lockdown period [41]. There was a 39.6% decrease in the 30-day incidence of trauma victims with a positive blood alcohol content, with a significant drop in the strict lockdown month of April with the closure of liquor shops [42]. The strict policies forcing the closure of liquor stores were beneficial in reducing alcohol-related traumas in India because trauma is a major cause of mortality and morbidity in developing countries. Knapen et al. noted a 23.3% decrease in alcohol-related ED visits in a Dutch trauma center during the first COVID-19 lockdown, with an even more pronounced 60% decrease in alcohol-related visits during the second COVID-19 lockdown which included an alcohol ban [43].

Ultimately, the effect of alcohol restrictions is subject to cultural, geographic, and hyperlocal variables. Almost half of Queens’ 2.2 million population is foreign-born, with over 138 languages spoken across the borough [44,45]. Alcohol use remains an important variable in preventable traumas. Changes seen in specific locations are not sufficient to inform public policy to restrict alcohol consumption. In NYC, we do not see the data to suggest that the relaxing of alcohol consumption led to an increase in alcohol-related TBI. Absolute numbers have to be examined as well, as we observed a decrease in alcohol-related TBI admissions during the lockdown but noticed an “increase” in alcohol positivity during the lockdown, as compared with post-lockdown. This “increase” in alcohol positivity could also be due to the decrease in non-alcohol-related traumas.

Our study is not without limitations. First, we defined any individuals who presented to the ED and were not screened for alcohol as alcohol-negative. This missing data may have led to the misclassification of patients who were alcohol positive at the time of ED presentation to be alcohol-negative. Second, our study focuses on New York only, but other states may have had different policies in place, and different start and end dates of the lockdown. This limits the external validity of our study to other states. Third, our study includes patients from one center in Queens. We can further increase the generalizability of our study by incorporating patients from other centers within the borough, and even expanding to incorporate patients from other boroughs within NYC.

## Conclusions

This study aimed to identify any differences between alcohol-positive patients presenting with TBI before, during, and after the COVID-19 pandemic. The results of this study demonstrate that the COVID-19 public policies put in place were not correlated with an increase in alcohol-related TBI admissions in Queens, New York. There was a decrease in total TBI admissions during the pandemic, in line with other major trauma centers, but despite the relaxation of alcohol consumption laws, there was no increase in alcohol positivity among TBI patients. Absolute numbers ought to be examined as well, as we observe a decrease in alcohol-related TBI admissions during the lockdown, but notice an “increase” in alcohol positivity during the lockdown, compared with post-lockdown. This “increase” in alcohol positivity could also be due to the decrease in non-alcohol-related traumas. Ultimately, the effect of alcohol restrictions is subject to cultural, geographic, and hyperlocal variables. This study highlights the complex interplay between public policies, alcohol use, and trauma during ongoing pandemic challenges.

## References

[1] McGraw C, Salottolo K, Carrick M (2021). Patterns of alcohol and drug utilization in trauma patients during the COVID-19 pandemic at six trauma centers. Inj Epidemiol.

[2] Matthews KR, Lakshmanan R, Kalakuntla N, Tallapragada N (2024). Personal rights over public health: anti-vaccine rhetoric in the Texas Legislature. Vaccine X.

[3] Chen G, Yao Y, Zhang Y, Zhao F (2024). The impact of risk perception and institutional trust on COVID-19 vaccine hesitancy in China. Hum Vaccin Immunother.

[4] Nasser R, Yadla S, Maltenfort MG (2010). Complications in spine surgery. J Neurosurg Spine.

[5] Onyeaka H, Anumudu CK, Al-Sharify ZT, Egele-Godswill E, Mbaegbu P (2021). COVID-19 pandemic: a review of the global lockdown and its far-reaching effects. Sci Prog.

[6] Seckman C (2023). The impact of COVID-19 on the psychosocial well-being of older adults: a literature review. J Nurs Scholarsh.

[7] Murthy P, Narasimha VL (2021). Effects of the COVID-19 pandemic and lockdown on alcohol use disorders and complications. Curr Opin Psychiatry.

[8] Jones EA, Mitra AK, Bhuiyan AR (2021). Impact of COVID-19 on mental health in adolescents: a systematic review. Int J Environ Res Public Health.

[9] Lechner WV, Laurene KR, Patel S, Anderson M, Grega C, Kenne DR (2020). Changes in alcohol use as a function of psychological distress and social support following COVID-19 related University closings. Addict Behav.

[10] Lange S, Probst C, Rehm J (2020). Coronavirus disease 2019 crisis and intentional injuries: now is not the time to erode alcohol control policies. Can J Public Health.

[11] Stockwell T, Andreasson S, Cherpitel C (2021). The burden of alcohol on health care during COVID-19. Drug Alcohol Rev.

[12] Kumar RG, Esterov D, Adams RS (2022). Changes in alcohol use and mood during the COVID-19 pandemic among individuals with traumatic brain injury: a difference-in-difference study. PLoS One.

[13] Katta-Charles S, Adams LM, Chiaravalloti ND (2023). Depression, anxiety, and suicidality in individuals with chronic traumatic brain injury before and during the COVID-19 pandemic: a National Institute on Disability, Independent Living, and Rehabilitation Research Traumatic Brain Injury Model Systems Study. Arch Phys Med Rehabil.

[14] Rajalu BM, Indira Devi B, Shukla DP (2022). Traumatic brain injury during COVID-19 pandemic-time-series analysis of a natural experiment. BMJ Open.

[15] Rault F, Terrier L, Leclerc A (2021). Decreased number of deaths related to severe traumatic brain injury in intensive care unit during the first lockdown in Normandy: at least one positive side effect of the COVID-19 pandemic. Acta Neurochir (Wien).

[16] Sohi I, Chrystoja BR, Rehm J, Wells S, Monteiro M, Ali S, Shield KD (2022). Changes in alcohol use during the COVID-19 pandemic and previous pandemics: a systematic review. Alcohol Clin Exp Res.

[17] Morrow EL, Patel NN, Duff MC (2021). Disability and the COVID-19 pandemic: a survey of individuals with traumatic brain injury. Arch Phys Med Rehabil.

[18] Dewan MC, Rattani A, Gupta S (2018). Estimating the global incidence of traumatic brain injury. J Neurosurg.

[19] Iaccarino C, Carretta A, Nicolosi F, Morselli C (2018). Epidemiology of severe traumatic brain injury. J Neurosurg Sci.

[20] Plurad D, Demetriades D, Gruzinski G (2010). Motor vehicle crashes: the association of alcohol consumption with the type and severity of injuries and outcomes. J Emerg Med.

[21] Weil ZM, Corrigan JD, Karelina K (2018). Alcohol use disorder and traumatic brain injury. Alcohol Res.

[22] Kim B, Rundle AG, Goodwin AT, Morrison CN, Branas CC, El-Sadr W, Duncan DT (2021). COVID-19 testing, case, and death rates and spatial socio-demographics in New York City: an ecological analysis as of June 2020. Health Place.

[23] Thomas AC, Campbell BT, Subacius H (2022). National evaluation of the association between stay-at-home orders on mechanism of injury and trauma admission volume. Injury.

[24] Laufer K, Petek K, Rakusa S, Rakusa M, Rakusa M, Cretnik A (2022). Traumatic brain injury during the SARS-CoV-2 pandemics in Slovenia: a single center study. J Clin Med.

[25] Foje NA, Waibel BH, Sheppard OO, Josef AP, Bauman ZM, Evans CH, Hamill ME (2023). Increase in alcohol use among the geriatric trauma population during the COVID-19 pandemic. Am Surg.

[26] Brown OS, Smith TO, Gaukroger AJ, Tsinaslanidis P, Hing CB (2022). Increased proportion of alcohol-related trauma in a South London major trauma centre during lockdown: a cohort study. Chin J Traumatol.

[27] Devarakonda AK, Wehrle CJ, Chibane FL, Drevets PD, Fox ED, Lawson AG (2021). The effects of the COVID-19 pandemic on trauma presentations in a Level One trauma center. Am Surg.

[28] Rhodes HX, Petersen K, Biswas S (2020). Trauma trends during the initial peak of the COVID-19 pandemic in the midst of lockdown: experiences from a rural trauma center. Cureus.

[29] Rudisill TM, Steinmetz L, Bardes JM (2023). Substance use in rural trauma patients admitted for motor vehicle injuries before and during the COVID-19 pandemic. Inj Epidemiol.

[30] Zhai M, Bono K, Zhang WW (2023). Drug and alcohol use in trauma patients before and during the COVID-19 pandemic. J Surg Res.

[31] Young KN, Yeates EO, Grigorian A (2021). Drug and alcohol positivity of traumatically injured patients related to COVID-19 stay-at-home orders. Am J Drug Alcohol Abuse.

[32] Sun A, Johnson D (2022). Characterization of traumatic injury during the early COVID-19 pandemic: results from a national healthcare database. Cureus.

[33] Meng L, Qiu H, Wan L (2020). Intubation and ventilation amid the COVID-19 outbreak: Wuhan's experience. Anesthesiology.

[34] Koçak Tufan Z, Kayaaslan B, Mer M (2021). COVID-19 and sepsis. Turk J Med Sci.

[35] Al Mazrouei SS, Saeed GA, Al Helali AA, Ahmed M (2020). COVID-19-associated encephalopathy: neurological manifestation of COVID-19. Radiol Case Rep.

[36] Fällmar D, Rostami E, Kumlien E (2022). The extent of neuroradiological findings in COVID-19 shows correlation with blood biomarkers, Glasgow coma scale score and days in intensive care. J Neuroradiol.

[37] Reza Bagheri S, Abdi A, Benson J (2021). The significant impact of coronavirus disease 2019 (COVID-19) on in-hospital mortality of elderly patients with moderate to severe traumatic brain injury: a retrospective observational study. J Clin Neurosci.

[38] Boehme AK, Doyle K, Thakur KT (2022). Disorders of consciousness in hospitalized patients with COVID-19: the role of the systemic inflammatory response syndrome. Neurocrit Care.

[39] van Hoving DJ, van Koningsbruggen C, de Man M, Hendrikse C (2021). Temporal changes in trauma according to alcohol sale restrictions during the South African national COVID-19 lockdown. Afr J Emerg Med.

[40] Reuter H, Jenkins LS, De Jong M, Reid S, Vonk M (2020). Prohibiting alcohol sales during the coronavirus disease 2019 pandemic has positive effects on health services in South Africa. Afr J Prim Health Care Fam Med.

[41] Soni P (2021). Effects of COVID-19 lockdown phases in India: an atmospheric perspective. Environ Dev Sustain.

[42] Abhilash KP, Paul AJ, Das S, Hazra D, Jain S, Dhinakar Arelly SP (2021). Changing pattern of trauma during the COVID-19 Pandemic. Med J Armed Forces India.

[43] Knapen FM, Laumer SJ, Van Osch FH, Barten DG (2022). The impact of the COVID-19 pandemic on alcohol-related emergency department visits in the Netherlands: the ALCOVID study. Drug Alcohol Rev.

[44] Babbel.com Babbel.com, GmbH LN (2023). Is Queens, New York, the most multilingual county in the world?. https://www.babbel.com/en/magazine/the-languages-of-queens-diversity-capital-of-the-world.

[45] (2023). U.S. Census Bureau QuickFacts: Queens County, New York. https://www.census.gov/quickfacts/fact/table/queenscountynewyork/PST045222.

